# Diseases of the Epididymis and Testicle*Abstract of Clinical Lecture Given in the Long Island College Hospital.

**Published:** 1916-03

**Authors:** Henry H. Morton

**Affiliations:** Clinical Professor of Genitourinary Diseases, Long Island College Hospital; Genitourinary Surgeon, Long Island College and Kings County Hospitals, and the Polhemus Memorial Clinic, Etc., Brooklyn, New York


### Abstracts and Selections.

### Diseases of the Epididymis and Testicle.*

BY HENRY H. MORTON, M. D., CLINICAL PROFESSOR OF GENITO-URINARY DISEASES, LONG ISLAND COLLEGE HOSPITAL; GENITO-URINARY SURGEON, LONG ISLAND COLLEGE AND KINGS COUNTY HOSPITALS, AND THE POLHEMUS MEMORIAL CLINIC, ETC., BROOKLYN, NEW YORK.

Epididymitis is more frequently seen than orchitis. Causes are gonorrhea, traumatism, passage of urethral instruments, or as a sequel to prostatectomy. The organism reach the epididymis through the vas deferens by means of its peristaltic action, which moves in either direction.

Causes of orchitis are syphilis, malignant disease, or metastasis during an attack of mumps.

*Abstract of Clinical Lecture Given in the Long Island College Hospital.

*Gonorrheal Epididymitis.*—Testicle red, swollen, tender, inflamed. The epididymis is enormously swollen and surrounds the testicle. A small quantity of hydrocele fluid is usually found. Discharge from urethra usually ceases while epididymis is affected and begins again as the inflammation subsides.

*Treatment.*—Preventive: Have patient wear suspensory and keep as quiet as possible. No urethral instruments should be passed and no forced injections given.

When present the patient should be put to bed, the scrotum supported with a handkerchief bandage, and continuous hot applications applied. Hot flaxseed poulties, or hot lead and opium work very well.

Cold is no longer used, as it is liable to leave a hard, tough infiltration of the epididymis, thereby causing sterility.

In moderately severe cases accompanied with great pain 20 per cent Guaiacol ointment covered with cotton and heat usually allays the suffering.

In recurring epididymitis or in very severe cases which do not respond to treatment the Hagner operation is indicated.

Where there is a great deal of hydrocele fluid present, aspiration of the fluid makes the patient comfortable.

Where the epididymis has been blocked as the result of inflammation and the patient sterile, the patent end of the epididymis may be transplanted into the testicle and a new channel for the exit of the spermatozoa is made. In about 50 per cent of cases operated the sterility is relieved.

*Tuberculosis of the Epididymis.*—Begins as a small painless lump, which gradually increases in size.

The epididymis is usually infected:

1. By hematogenous infection.
2. By extension from higher up; vesicles, prostate, kidneys.

Later the testicle becomes infected, and breaks down and becomes filled with pus.

The focus may remain latent for a long time, and then be lighted up by some traumatism.

*Treatment.*—Epididymectomy is the operation of choice when the testicle is not involved, but when the testicle is affected castration is indicated.

After the patient recovers from the operation he should be instructed to lead an out-of-door life, as ordered in the case of any other tuberculous individual.

TREATMENT.—"Why are you hard on Dr. Bones?"

"When I broke my arm he pulled my leg to effect a cure."—*Valley View Sun.*

---

Doctor—"I'll examine you carefully for $10."

Weary Dreary—"All right, an' if you find it, give me half."—*Eastern Star.*

---

What type of parent would a child select for itself? Eugenic development is not possible along the lines satisfactory for breeding animals.—*Medical Review of Reviews.*

